# Inhibition of oleandrin on the proliferation show and invasion of osteosarcoma cells in vitro by suppressing Wnt/β-catenin signaling pathway

**DOI:** 10.1186/s13046-015-0232-8

**Published:** 2015-10-06

**Authors:** Yunlong Ma, Bin Zhu, Xiaoguang Liu, Huilei Yu, Lei Yong, Xiao Liu, Jia Shao, Zhongjun Liu

**Affiliations:** Department of Orthopaedics, Peking University Third Hospital, North Garden Street No. 49, Haidian District, Beijing, 100191 People’s Republic of China

**Keywords:** Osteosarcoma, Oleandrin, Proliferation, Invasion, Wnt/β-catenin signaling, Antitumor activity

## Abstract

**Background:**

Osteosarcoma (OS) is a high-grade bone sarcoma with early metastasis potential, and the clinical chemotherapy drugs that are currently used for its treatment have some limitations. Recently, several studies have reported the selective antitumor effect of oleandrin on various tumor cells. In this study, we aimed to evaluate the effects and underlying mechanisms of oleandrin on OS cells.

**Methods:**

The effect of oleandrin on the proliferation, morphology, and apoptosis of U2OS and SaOS-2 cells were analyzed in vitro. The activity of the Wnt/β-catenin signaling pathway was determined using a dual luciferase assay. Semi-quantitative RT-PCR and western blot assays were performed to evaluate the mRNA and total protein expression of the downstream target genes. Changes of β-catenin in intracellular localization were also explored using a western blot after separating the nucleus and cytoplasm proteins. The MMP-2 and MMP-9 enzymatic activities were determined using gelatin zymography.

**Results:**

Oleandrin significantly inhibited the proliferation and invasion of OS cells in vitro, and induced their apoptosis. After treatment with oleandrin, the TOP/FOP flash ratio in OS cells was noticeably decreased, which indicated that the Wnt/β-catenin signaling pathway was repressed. The expression of related Wnt target genes and total β-catenin was downregulated, and a reduced nuclear β-catenin level by oleandrin was observed as well. In addition, oleandrin suppressed the activities of MMP-2 and MMP-9.

**Conclusions:**

Oleandrin, in vitro, exerted a strong antitumor effect on human OS cells by suppressing the Wnt/β-catenin signaling pathway, which interfered with the proliferation and invasion of OS cells, as well as induced cells apoptosis. Moreover, the expression and activities of MMP-2 and MMP-9 were downregulated by oleandrin, which contributed to the cells’ lower invasiveness.

## Background

Osteosarcoma (OS) is a high-grade malignant bone tumor caused by genetic and epigenetic changes that interrupt osteoblast differentiation from mesenchymal stem cells with a high potential for local destruction and distant metastasis [1, 2]. OS mainly emerges in regions of active bone growth, such as the knee joint, lower femur and upper tibia, with a high incidence in adolescents. The treatment for OS, which combines surgery with neoadjuvant and adjuvant chemotherapy, has developed rapidly [3]. Although the use of multi-drug chemotherapy has greatly increased the 5-year survival rate of patients, approximately 30 % of patients experience relapse or metastasis. Importantly, the 5-year survival rate of OS patients with pulmonary metastasis is extremely poor, at 10–20 % [4]. Moreover, a variety of problems also exist with the current therapies, such as chemotherapy tolerance and side effects. This context limits the application of clinical chemotherapy drugs. Therefore, the identification a new drug with selective cytotoxicity to tumor cells is urgently needed.

Cardiac glycosides, a type of traditional drug that is used to treat cardiac insufficiency, were recently reported to have an antitumor effect on many tumor cells including breast cancer, lung cancer as well as leukemia [5–7]. Oleandrin is a polyphenolic cardiac glycoside derived from the leaves of Nerium oleander, which has been found to have anti-proliferative effects on tumor cells [5]. The evidence indicates that oleandrin could be a perfect alternative substance due to its selective antitumor and chemoradiation sensitization properties [8]. Moreover, oleandrin recently underwent a Phase I clinical trial as a novel drug for anticancer therapy in patients with refractory solid tumors [9].

There are some reports about the antitumor mechanisms of oleandrin. Cardiac glycosides, such as oleandrin, are known to inhibit the Na^+^, K^+^-ATPase pump, resulting in a consequent increase of calcium influx in the heart muscle [10]. Previous studies reported that oleandrin inhibited the proliferation and induced the apoptosis of cells due to an increase in intracellular Ca^2+^ via the inhibition of Na^+^, K^+^-ATPase [11]. Oleandrin inhibited the export of fibroblast growth factor-2 through membrane interactions and Na^+^, K^+^-ATPase activity in prostate cancer cells [12]. Oleandrin also induced apoptosis in human leukemia cells through the dephosphorylation of Akt and expression of Fas L, as well as by altering the membrane fluidity [8]. In addition, it suppressed the activation of NF-kB and induced Fas expression and autophagosome formation in tumor cells. The inhibition of Akt phosphorylation and the increase of MAPK expression were also demonstrated in response to oleandrin. The results of these studies have indicated an impending injury and the death of tumor cells following oleandrin treatment. However, no studies have described the antineoplastic activity of oleandrin on osteosarcoma cells and the related mechanisms.

Recent studies have reported the important effect of the Wnt/β-catenin signaling pathway in tumorigenesis, bone development and stem cell biology [13, 14]. Additionally, the role of Wnt/β-catenin signaling pathway in the occurrence of osteosarcoma has also been brought to the forefront. The Wnt/β-catenin pathway is activated when a Wnt ligand binds to its coreceptor complex, which contains a Frizzled family member and low-density lipoprotein receptor-related protein 5/6 (LRP-5/6). Subsequently, disheveled (Dsh) is activated and transmits signals from the receptor to the APC/axin/GSK3 destruction complex to suppress the phosphorylation of β-catenin by GSK-3β [15, 16]. Thus, unphosphorylated β-catenin accumulates in the cytoplasm and translocates into the nucleus, interacts with the T-cell factor/lymphocyte enhancer factor (TCF/LEF) family of transcription factors and activates downstream target genes, including c-myc, cyclin D1, survivin and matrix metalloproteinase (MMPs), which regulate cell cycle and apoptosis, as well as metastasis [17].

In this study, we aimed to evaluate the effect of oleandrin on OS cell lines in vitro and to investigate the underlying mechanism involved in this process.

## Methods

### Cell lines and culture

U2OS and SaOS-2 cell lines, derived from the China Infrastructure of Cell Line Resources (Chinese Academy of Medical Sciences, Beijing, China), were cultured in McCoy’s 5A (HyClone Laboratories of Thermo Scientific, Logan, UT, USA) and Dulbecco’s modified eagle medium (DMEM, HyClone Laboratories of Thermo Scientific, Logan, UT, USA), respectively, which were supplemented with 10 % fetal bovine serum (FBS, HyClone Laboratories of Thermo Scientific, Logan, UT, USA), 100 units/ml penicillin and 100 μg/ml streptomycin (Gibco, Life Technologies, Inc., Grand Island, NY) in a humidified incubator containing 5 % CO_2_ at 37 °C. Oleandrin was purchased from Sigma-Aldrich Chemical Co. (St. Louis, CA, USA), and the purity was approximately 99 %, as analyzed by HPLC. It was dissolved in 100 % dimethyl sulfoxide (DMSO, Sigma-Aldrich Chemical Co., St. Louis, CA, USA) and diluted with medium. As a vehicle control, cultured cells were incubated in medium containing DMSO at a final concentration of less than 0.1 %.

### Cell counting kit-8 (CCK-8) proliferation assay

U2OS or SaOS-2 cells were seeded in a 96-well dish at a final density of 5 × 10^3^ (U2OS) or 1 × 10^4^ (SaOS-2) cells/well and were incubated for 24 h. The cells were then treated with oleandrin at increasing concentrations (25, 50, 75, and 100 nM) and the control medium for 24, 48 and 72 h. Thereafter, 10 μl CCK-8 (Dojindo Laboratories, Kumamoto, Japan) was added to each well and incubated for another 3 h. The absorption at 450 nm was determined for each sample using an automatic ELISA plate reader. Five replicate wells were used for each treatment, and the experiments were repeated three times. Cell viability (%) = [OD (treatment)-OD (blank)]/[OD (control)-OD (blank)] × 100 %.

### Colony formation assay

U2OS and SaOS-2 cells were seeded into 12-well plates at a density of 100 cells/well. After adherence at 37 °C in a 5 % CO_2_ humidified oven for 24 h, the cells were treated with 25 nM and 50 nM oleandrin and the control medium at 37 °C for 10 days, during which the medium containing the corresponding concentrations of oleandrin was refreshed every 2 days. Ten days later, the colonies were fixed with 4 % paraformaldehyde and stained with 0.5 % crystal violet. The number of colonies in each well was counted under an inverted microscope (Leica, Frankfurt, Germany).

### DAPI staining

U2OS and SaOS-2 cells were seeded in 24-well plates and treated with the control or 25 and 50 nM oleandrin for 24 h. The cells were rinsed three times with PBS, and Triton X-100 was added to break the cell membrane integrity for 15 min. Then, DAPI (Beyotime, Shanghai, China) was added for another 10 min of incubation in the dark. The cell nuclei were observed and photographed at 400× magnification under a fluorescence microscope (Leica DM3000, Frankfurt, Germany).

### Apoptosis analysis by flow cytometry (FCM)

U2OS and SaOS-2 cells were seeded into 6-well plates and adhered overnight. When a confluence of 70–80 % was reached, both cell lines were exposed to 50 nM of oleandrin for 0, 24 and 48 h. Subsequently, the cells were collected and re-suspended in 500 μl of 1× binding buffer. Five microliters of annexin V-FITC and 5 μl of propidium iodide (PI) were added to all of the samples, which were then incubated at room temperature for 5 min in the dark according to the manufacturer’s protocol from the Annexin V-FITC apoptosis detection kit (BioVision Co, California, USA). The fluorescence intensity of the cells was immediately analyzed by flow cytometry.

### Wound-healing assay

U2OS and SaOS-2 cells were seeded in a 6-well plate and incubated for 24 h. A pipette tip was used to scratch three perpendicular wounds into a cross shape, and the wells were washed twice with PBS to remove the detached cells. U2OS and SaOS-2 cells were treated with 25 nM oleandrin for a corresponding amount of time. Oleandrin were diluted with medium containing 2 % FBS to eliminate the influence of cell proliferation. The wounds were photographed at each time point using an inverted microscope (Leica). The distance migrated was calculated by dividing the distance at the time point by the distance at the beginning. For each experiment, a total of 5 wounds were measured per group, and the results were analyzed using Image J Software.

### Transwell invasion assay

U2OS and SaOS-2 cells were starved in serum-free medium for 24 h and then re-suspended in serum-free medium with 25 nM oleandrin and the control medium. Then, 5 × 10^4^ cells per well were added to the upper chamber pre-coated with Matrigel (diluted 4-fold with PBS, BD Biosciences, Franklin Lakes, NJ), while the lower chamber was filled with 500 μl of complete culture medium containing 10 % FBS as a chemo-attractant source. After 24 h of incubation at 37 °C, the cells that had invaded the lower surface of the membrane were fixed with 75 % ethanol and stained with crystal violet. Using light microscopy, 5 random fields were selected, and the cells in each field were counted under high magnification (200×).

### Gelatin zymography assay

U2OS cells were seeded in 6-well plates and incubated with 1 ml of McCoy’s 5A medium containing oleandrin for 24 h after adherence. Then, the supernatant was collected and the protein concentration was determined using the BCA Protein Assay Kit (Applygen Technologies Inc., Beijing, China) according to the manufacturer’s protocol. All samples were diluted in 2 × SDS-PAGE non-reducing buffer (4 % SDS, 100 mM Tris–HCl pH6.8, 20 % glycerol and 0.02 % bromophenol blue), and the mixtures were separated on a SDS-PAGE gel (10 % separation gel containing gelatin). The following processes were performed according to the protocol from the MMP Zymography Assay Kit (Applygen Technologies Inc., Beijing, China). The gel was scanned by a gel documentation system (Bio-Rad Co., Nanjing, China).

### Semi-quantitative reverse transcription polymerase chain reaction (RT-PCR)

For PCR analysis, U2OS cells were treated with 50 nM oleandrin for 0, 24 and 48 h. Total RNA was extracted using TRIzol reagent (Invitrogen, Carlsbad, CA) according to the manufacturer’s protocol. Ten micrograms of RNA were reverse transcribed into cDNA using GoScript™ Reverse Transcription System (Promega, Southampton, UK). The cDNA was added to a 2 × Taq PCR MasterMix (Tiangen Biotech Co., LTD, Beijing, China) containing 10 pmol/L of each of the corresponding primer pairs. The detailed information of the primers used in this analysis was listed in Table 1. PCR amplification was performed with corresponding cycles of 94 °C for 3 min, 94 °C for 30 s, at the annealing temperature for 30 s, 72 °C for 1 min and 72 °C for 5 min. The PCR products were separated on 1 % agarose gels and were stained with ethidium bromide. The gels were scanned under a gel documentation system (Bio-Rad Co.). β-actin was used as an internal reference to verify equal concentrations of cDNA in each sample.

**Table 1:** The details of primers in semi-quantitative RT-PCR

Gene	Primer sequence (5’ to 3’)	Annealing temperature (°C)	Cycle number
c-Myc	F: CCACACATCAGCACAACTACG	57	30
	R: CCGCAACAAGTCCTCTTCAG		
cyclin D1	F: TCGGGAGAGGATTAGGTTCC	57	30
	R: GTCACTGGATGGTTTGTTGG		
survivin	F: GTCCCTGGCTCCTCTACTGTT	57	30
	R: GATGTGAAGGTTGGGCTGAC		
MMP-2	F: GACCACAGCCAACTACGATG	59	30
	R: CACAGTCCGCCAAATGAAC		
MMP-9	F: CATCGTCATCCAGTTTGGTGT	57	32
	R: AGGGTTTCCCATCAGCATT		
β-actin	F: AGCGAGCATCCCCCAAAGTT	59	25
	R: GGGCACGAAGGCTCATCATT		

### Western blot analysis

For protein expression analysis, U2OS cells were seeded in 100-mm dishes and treated with 50 nM oleandrin for 0, 24 and 48 h. Total proteins were extracted by RIPA lysis buffer (Applygen Technologies Inc., Beijing, China). Cell nuclear and cytoplasm proteins were extracted separately using the NE-PER™ Nuclear and Cytoplasm Extraction Reagents (Pierce Biotechnology Inc., Rockfold, USA) according to the manufacturer’s protocol. The protein concentration was detected using the BCA method. All the samples were mixed with 5 × sodium dodecyl sulfate (SDS) loading buffer (1:4), boiled for 5 min and stored at −80 °C for later use. The same amount of protein was separated on a discontinuous SDS-PAGE gel (8–15 % separation gel) and transferred to a nitrocellulose membrane after electrophoresis. The membranes were blocked with 5 % BSA in TBS containing 0.05 % Tween 20 for 2 h and were incubated with corresponding rabbit primary antibodies overnight at 4 °C. The antibodies included β-catenin (1:1000, Santa Cruz Biotechnology, USA), cyclin D1, survivin, c-myc (1:1000, Cell Signaling Technology, Boston, Massachusetts, USA), MMP-9, lamin A/C, α-tubulin (1:1000, Abcam, Cambridge, MA, USA) and GAPDH (1:2000, Tiangen Biotech Co., LTD, Beijing, China). Among them, lamin A/C and α-tubulin were used as internal controls for the nuclear and cytoplasm proteins, respectively. GAPDH were used as an internal control for the total protein. The secondary antibody was an IRDye 800CW conjugated goat (polyclonal) anti-rabbit IgG secondary antibody (1:10000, LI-COR, Nebraska, USA). The fluorescent bands were visualized with an Odyssey infrared imaging system (LI-COR), and the gray values were analyzed using Odyssey V3.0 software.

### Dual luciferase assay

The dual luciferase assay was performed with the dual-luciferase®reporter assay system (Promega Corp., Madison, WI). All reagents were prepared as described by the manufacturer of the TCF Reporter Plasmid Kit (Millipore Corp., MA, USA). After the transfection complex was formed with FOP DNA, pRL-TK Vector renilla (Promega Corp., Madison, WI) and 50 μl of DNA Lipofectamine™ 2000 (Invitrogen, Carlsbad, CA), the cells were seeded into 96-well plates with 100 μl/well. U2OS cells were divided into four groups: oleandrin (time): 50 nM oleandrin for 0, 24 and 48 h; oleandrin (concentration): 0, 25 and 50 nM oleandrin for 24 h, LiCl + oleandrin (time) and LiCl + oleandrin (concentration). For the LiCl groups, the cells were pretreated with 20 mM LiCl for 6 h to activate the Wnt signaling pathway. After the cells were lysed with PLB for 15 min, firefly luciferase reagent LARII (100 μl) and Stop & Glo Reagent (100 μl) (Promega Corp., Madison, WI) were added. The OD values of the TOP flash and the FOP flash were detected and the TOP/FOP ratio reflected the activity of the Wnt/β-catenin signaling pathway.

### Statistical analysis

The data were presented as the means ± standard deviation (SD) and were analyzed with PASW statistics 18 software. A value of *P* < 0.05 was considered statistically significant. Comparisons of two or more data sets were analyzed using one-way analysis of variance (ANOVA), and data with more than two variables were analyzed using two-way repeated-measures ANOVAs with post hoc Tukey’s analysis.

## Results

### The viability and proliferation of OS cells were suppressed by oleandrin

The CCK-8 assay was performed to evaluate the anti-proliferative effect of oleandrin on U2OS and SaOS-2 cells. Using various concentrations of oleandrin to treat cells for different times, both cell lines exhibited significantly different viabilities with a decreasing trend of concentration- and time- dependency (1a, b). For U2OS, the administration of 25 nM oleandrin decreased the cell viability without a significant difference at 24 h (*P* > 0.05), but with a significant difference at 48 h (*P* < 0.01). However, the viability of cells was reduced significantly after treatment with 50 nM oleandrin for 24 h (*P* < 0.01) and 48 h (*P* < 0.01). Subsequently, only a few cells remained at 72 h post-treatment. For SaOS-2, however, both 25 nM and 50 nM oleandrin significantly decreased cell viability after treatment for 24 h (*P* < 0.01) and 48 h (*P* < 0.01). Based on these results, we selected 25 nM or 50 nM oleandrin to treat the cells for 24 h and 50 nM oleandrin to treat cells for 24 h or 48 h in the following experiments.

**Fig. 1:**
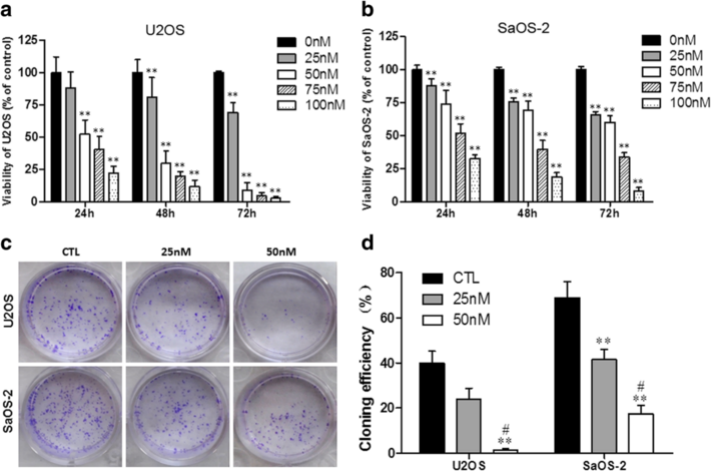
The inhibiting effect of oleandrin on OS cell proliferation. **a**/**b** The viability of U2OS (**a**) and SaOS-2 (**b**) cells after treatment with various concentrations of oleandrin for varying times. **c** A macrograph of the clone formation of the U2OS and SaOS-2 cells. **d** Cloning efficiency (%) of U2OS and SaOS-2 cells. *n* = 3. Mean ± SD. ^**^ *P* < 0.01, vs. control group (CTL). ^#^ *P* < 0.05, vs. 25 nM

The influence of oleandrin on the colony forming abilities of OS cells was also observed by performing plate clone formation assays. Both U2OS and SaOS-2 cells were isolated separately and cloned as described in the Methods section. After treatment with 25 nM and 50 nM oleandrin for 24 h, the U2OS and SaOS-2 colonies were significantly reduced (Fig. 1c). The cloning efficiencies of U2OS at 25 nM and 50 nM compared with the control were 24.0 % and 1.5 % vs. 39.8 % (25 nM or 50 nM vs. control: *P* = 0.207 or *P* = 0.002; 25 nM vs. 50 nM: *P* = 0.019), respectively (Fig. 1d). Correspondingly, the cloning efficiencies of SaOS-2 at 25 nM and 50 nM compared with the control were 41.5 % and 17.5 % vs. 69.0 % (25 nM or 50 nM vs. control: *P* = 0.005 or *P* = 0.000; 25 nM vs. 50 nM: *P* = 0.011), respectively (Fig. 1d).

### The morphology of OS cells was changed by oleandrin treatment

**Fig. 2:**
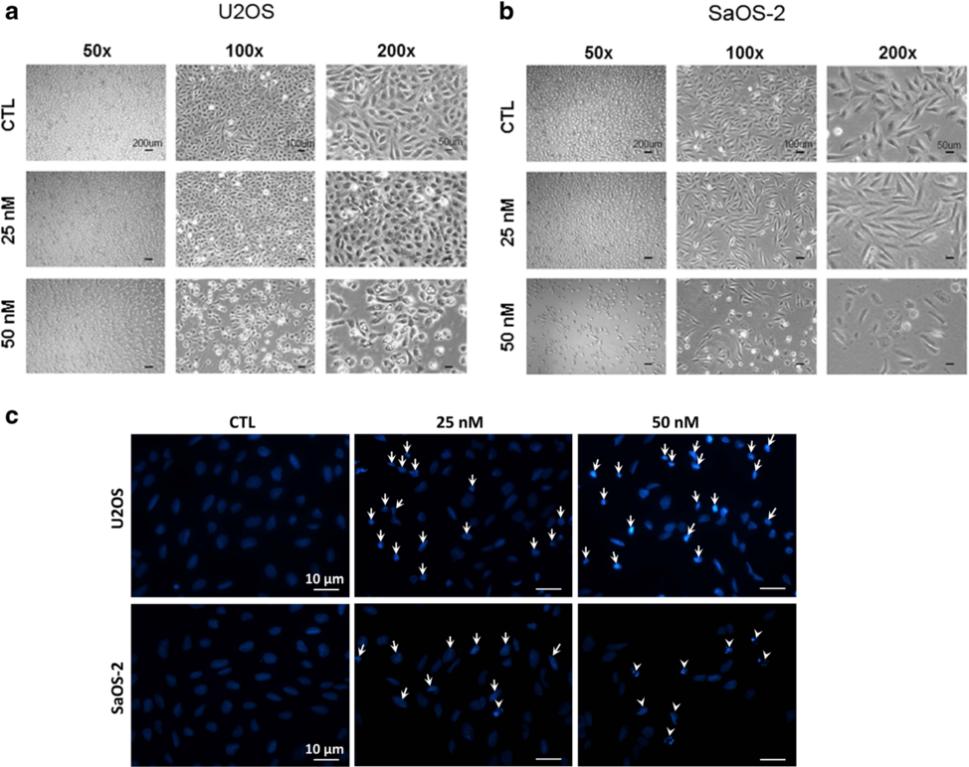
The changes of the cell morphologies and cell nuclei caused by increasing concentrations of oleandrin. (**a**/**b**) The morphology of U2OS (**a**) and SaOS-2 (**b**) cells was observed with an optical microscope at 50×, 100× and 200× magnification. **c** Nuclei staining of U2OS and SaOS-2 cells was performed with DAPI and was photographed at 400× magnification (karyopyknosis: arrow pointing; karyorrhexis: arrowhead pointing)

After treatment with 25 nM and 50 nM oleandrin for 24 h, U2OS and SaOS-2 cells were observed by an optical microscope at a low magnification (50×) to a high magnification (100× and 200×). After exposure to oleandrin, the number of U2OS and SaOS-2 cells gradually reduced at low magnification. At high magnification, we also observed that both cell lines presented typical apoptotic morphological changes, which included the irregularities in the cell surfaces and the vesicles in the cytoplasm after exposure to 25 nM and 50 nM oleandrin (Fig. 2a, b).

### Oleandrin induced OS cells apoptosis

Dye 4’-6-diamidino-2-phenylindole (DAPI) staining is a classic method to reflect the morphological changes of the cell nucleus in apoptosis. Fig. 2c shows that without oleandrin, the cell nuclei of U2OS and SaOS-2 cells are dispersed uniformly. However, after exposure to the drug, many OS cell nuclei became pyknotic and underwent karorrhexis and karyolysis.

**Fig. 3:**
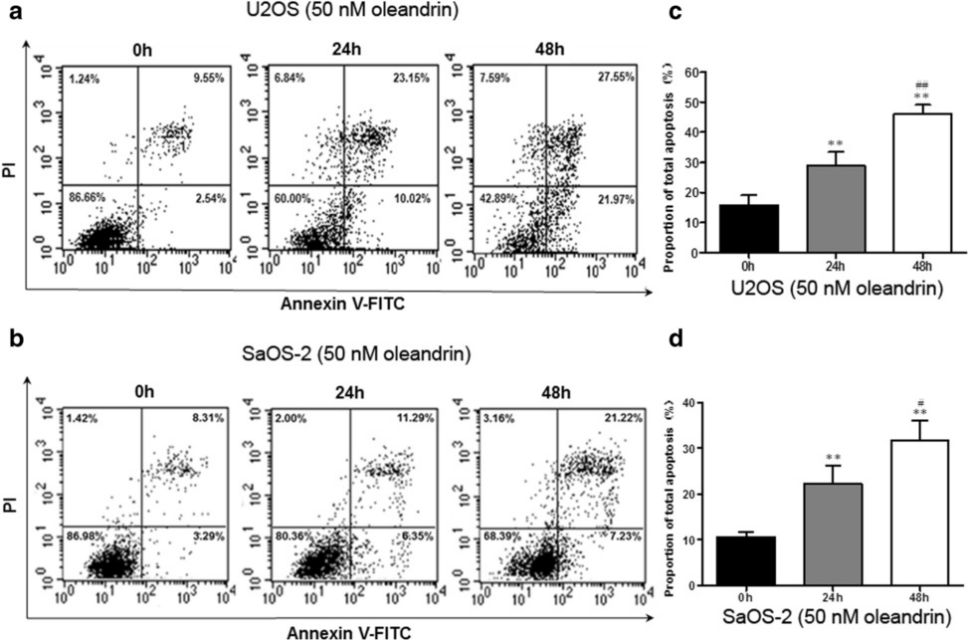
The apoptosis analysis of U2OS and SaOS-2 cells by flow cytometry (FCM). **a**/**b** The total apoptotic rates of U2OS (**a**) and SaOS-2 (**b**) cells after treatment with 50 nM oleandrin for 0, 24, and 48 h. **c**/**d** The quantitative results of the apoptosis analysis of U2OS (**c**) and SaOS-2 (**d**) cells. *n* = 3. Mean ± SD. ^**^ *P* < 0.01 vs. 0 h. ^#^ *P* < 0.05, ^##^ *P* < 0.01 vs. 24 h

Next, we detected FCM apoptosis following Annexin V-FITC and PI double staining to confirm this phenomenon. After treatment with 50 nM of oleandrin, the total number of apoptosed cells in both the U2OS and SaOS-2 cell lines increased significantly (Fig. 3a, b). The apoptosis rates of U2OS cells at 0, 24 and 48 h were 15.8 %, 29.0 % and 46.0 %, respectively (24 or 48 vs. 0 h: *P* = 0.005 or *P* = 0.000; 24 vs. 48 h: *P* = 0.001) (Fig. 3c). Similarly, the apoptosis rates of the SaOS-2 cells were 10.6 %, 22.2 % and 31.8 %, respectively (24 or 48 vs. 0 h: *P* = 0.007 or *P* = 0.000; 24 vs. 48 h: *P* = 0.015) (Fig. 3d).

### Oleandrin suppressed the migration and invasion of U2OS and SaOS-2 cells

**Fig. 4:**
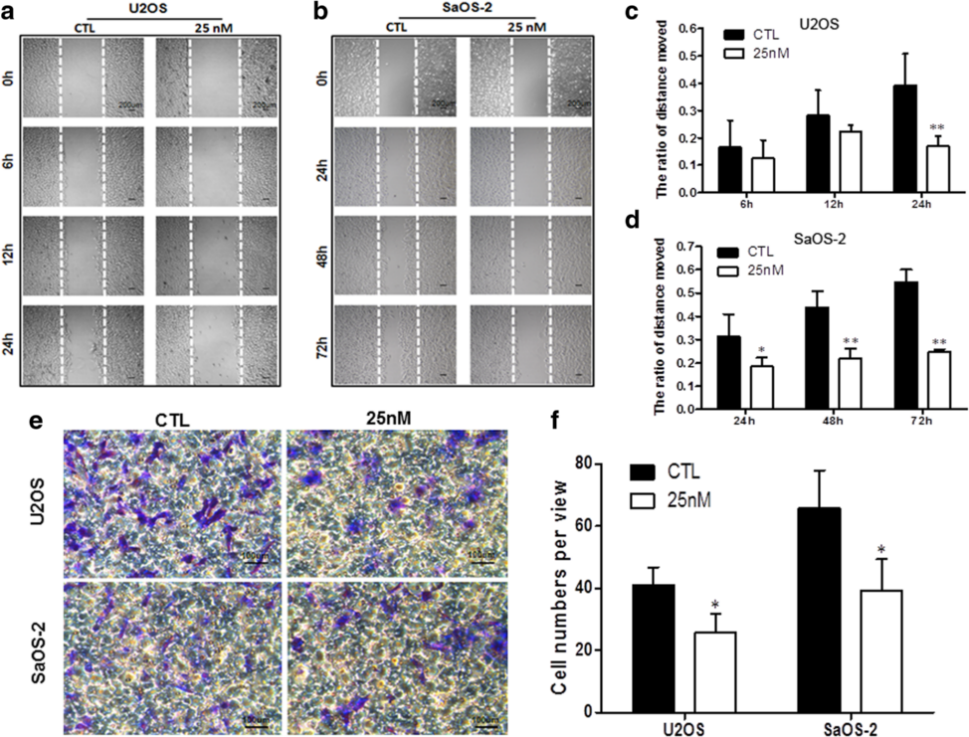
The changes of OS cell migration and invasion abilities after treatment with oleandrin. **a**/**b** The migration of U2OS and SaOS-2 cells before and after treatment of U2OS cells (**a**, for 6, 12 and 24 h) and SaOS-2 cells (**b**, for 24, 48 and 72 h) with 25 nM oleandrin, was determined by a wound healing assay. **c**/**d** A graphical representation of the average distance moved by U2OS (**c**) and SaOS-2 (**d**) cells in the oleandrin-treated and control groups. **e** The invasiveness of U2OS and SaOS-2 cells was observed by a transwell invasion assay after treatment with 25 nM oleandrin for 24 h and was compared to the control group. **f** The number of cells that invaded the substratum of the membrane per view under a 200× magnification. *n* = 3. Mean ± SD. ^*^ *P* < 0.05, ^**^ *P* < 0.01, vs. control group (CTL)

Given the cytotoxic activity of oleandrin at high concentrations, we used a low concentration (25 nM) to evaluate the effect of oleandrin on cell migration and invasion in vitro with wound healing and transwell invasion assays, respectively. In our pre-experiments, the migration rate of U2OS was greater than that of SaOS-2, and after treatment with oleandrin for nearly 48 h, the scratches in the control of the U2OS cell line had already closed while the scratches in the control of the SaOS-2 cell line had not (data not shown). Therefore, we selected the treatment length for U2OS to be 6, 12 and 24 h and the treatment length for SaOS-2 to be 24, 48 and 72 h. The results showed that with an increased treatment time, the migration capabilities of both cell lines were suppressed (Fig. 4a, b). The ratio of the distance migrated in the control group compared with the 25 nM oleandrin group in U2OS cells at 6, 12 and 24 h was 16.6 % vs. 12.6 % (*P* = 0.482), 28.2 % vs. 22.4 % (*P* = 0.213) and 39.3 % vs. 17.1 % (*P* = 0.003), respectively (Fig. 4c). Meanwhile, in the SaOS-2 cells, the corresponding results at 24, 48 and 72 h were 31.4 % vs. 18.5 % (*P* = 0.023), 43.8 % vs. 21.9 % (*P* = 0.000) and 54.7 % vs. 24.8 % (*P* = 0.000), respectively (Fig. 4d).

Consistent with the wound healing assay, the results of the transwell invasion assay indicated that OS cells that invaded from the Matrigel into the substratum of the membrane were significantly decreased after treatment (Fig. 4e). The numbers in the substratum of the membrane per view under high magnification (200×) in the control group compared with the 25 nM oleandrin group of U2OS cells were 41.1 ± 5.7 vs. 25.8 ± 6.1 (*P* = 0.033), and the corresponding numbers of SaOS-2 cells were 65.8 ± 12.3 vs. 39.4 ± 10.0 (*P* = 0.045) (Fig. 4f).

### Oleandrin suppressed the activity of Wnt/β-catenin signaling pathway

**Fig. 5:**
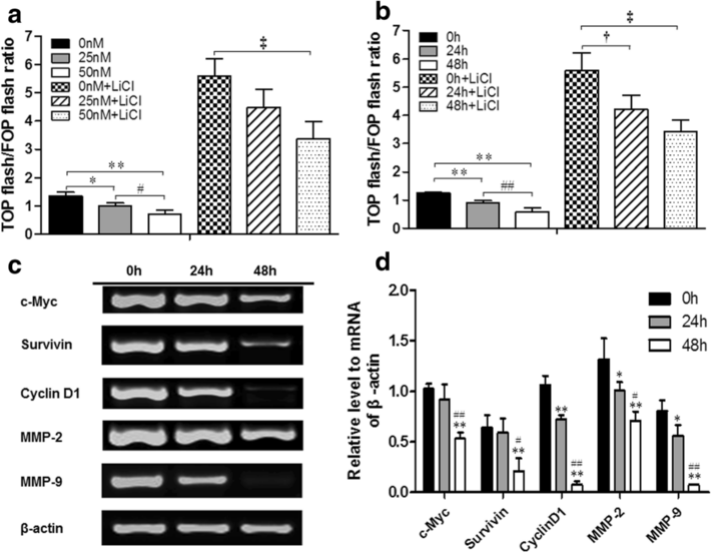
Changes in the Wnt/β-catenin signaling activities of U2OS and the mRNA expression levels of related downstream genes in this pathway. (**a**/**b**) With or without pretreatment with 20 μM LiCl, the TOP/FOP flash ratios were detected using a dual-luciferase assay in U2OS cells after treatment with 0, 25 and 50 nM of oleandrin for 24 h (**a**), or 50 nM of oleandrin for 0, 24 and 48 h (**b**), respectively. *n* = 3, Mean ± SD. ^*^ *P* < 0.05, ^**^ *P* < 0.01, compared to the control group without LiCl in each intervention condition (0 nM group or 0 h group); ^#^ *P* < 0.05, ^##^ *P* < 0.01, in the absence of LiCl, 25 nM group v.s. 50 nM group, 24 h group vs. 48 h group; ^†^ *P* < 0.05, ^‡^ *P* < 0.01, compared to the control group with LiCl in each intervention condition (0 nM group or 0 h group). **c** The gel electrophoresis of c-myc, survivin, cyclin D1, MMP-2, MMP-9 and β-actin after amplification. **d** The semi-quantitative relationship between the mRNA expression levels of related target genes and β-actin in U2OS cells according to the results of gel electrophoresis in RT-PCR after treatment with 50 nM of oleandrin for 0, 24 and 48 h. ^*^ *P* < 0.05, ^**^ *P* < 0.01, compared to the 0 h group. ^#^ *P* < 0.05, ^##^ *P* < 0.01, compared to the 24 h group

Previous studies reported that the abnormal activation of the Wnt signaling pathway plays an important role in OS pathogenesis. In this study, we explored whether oleandrin had an effect on the Wnt/β-catenin signaling pathway, and a dual-luciferase assay was used to evaluate this effect in U2OS cells. Fig. 5a shows that without LiCl, an inhibitor of GSK-3β, oleandrin was able to suppress the activities of Wnt/β-catenin signaling by downregulating the TOP/FOP flash ratio in a concentration-dependent manner (25 nM or 50 nM vs. control: *P* = 0.017 or *P* = 0.001, 25 nM vs. 50 nM: *P* = 0.043). In addition, after pretreatment with LiCl, the TOP/FOP flash ratio first increased but then declined in a concentration-dependent manner after oleandrin treatment (25 nM or 50 nM vs. control: *P* = 0.073 or *P* = 0.005, 25 nM vs. 50 nM: *P* = 0.070). Similarly, Fig. 5b also shows that oleandrin could downregulate the TOP/FOP flash ratio in a time-dependent manner with LiCl (24 or 48 h vs. 0 h: *P* = 0.004 or *P* = 0.000, 24 vs. 48 h: *P* = 0.005) or without LiCl (24 or 48 vs. 0 h: *P* = 0.017 or *P* = 0.002, 24 vs. 48 h: *P* = 0.120).

### Oleandrin downregulated the target gene expression of the Wnt/β-catenin pathway at both the mRNA and protein levels

**Fig. 6:**
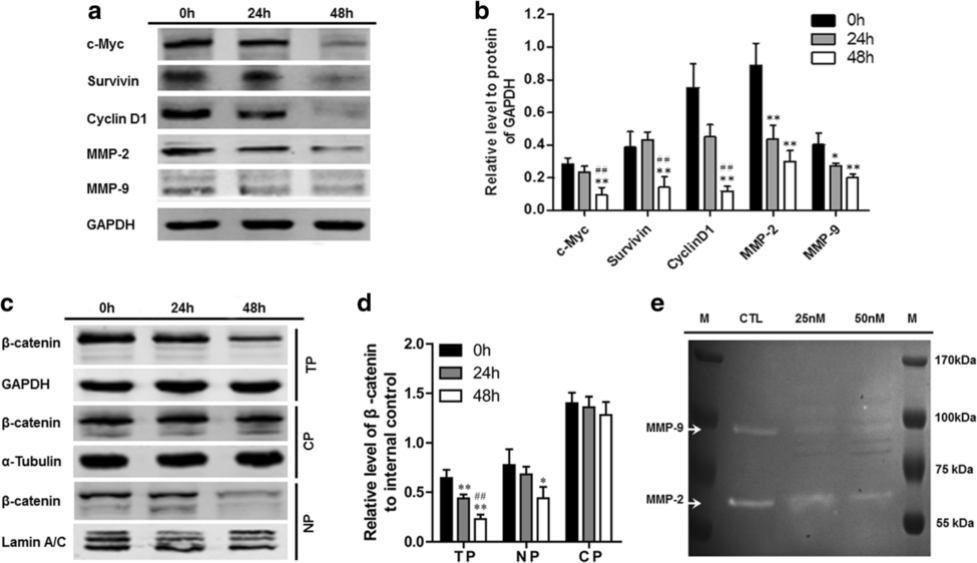
Western blotting and gelatin zymography. **a** The protein expression levels of the relevant downstream molecules in the Wnt/β-catenin pathway after the treatment of U2OS cells with 50 nM oleandrin for 0, 24 and 48 h as determined by western blotting. **b** The semi-quantitative results of the relevant downstream molecules in the Wnt/β-catenin pathway relative to the GAPDH protein. ^*^ *P* < 0.05, ^**^ *P* < 0.01, compared to the 0 h group. ^#^ *P* < 0.05, ^##^ *P* < 0.01, compared to the 24 h group. **c** Representative electrophoretograms of the total, nuclear and cytoplasmic β-catenin levels after treatment with 50 nM of oleandrin for 0, 24 and 48 h using western blotting. **d** The semi-quantitative results of the total, nuclear and cytoplasmic β-catenin levels based on electrophoretograms from the western blot analysis. ^*^ *P* < 0.05, ^**^ *P* < 0.01, compared to the 0 h group. ^#^ *P* < 0.05, ^##^ *P* < 0.01, compared to the 24 h group. (**e**) The MMP-2 and MMP-9 activities after treatment with 25 and 50 nM of oleandrin as detected by a gelatin zymography assay. CP: cytoplasmic protein; NP: nuclear protein; TP: total protein

To study the effect of oleandrin on the Wnt/β-catenin signaling pathway, we detected the mRNA and total protein expression changes of the downstream target genes, which included c-myc, survivin, cyclin D1, MMP-2 and MMP-9, using semi-quantitative RT-PCR and western blot assays. As we expected, the results of the RT-PCR showed that oleandrin significantly downregulated the mRNA levels of these genes to different degrees dependent on treatment time (Fig. 5c, d). In accordance with the RT-PCR results, after treatment with oleandrin for 24 and 48 h, the protein expression of the target genes was reduced, which indicated that oleandrin had a remarkable inhibiting effect on the downstream molecules of the Wnt/β-catenin signaling pathway (Fig. 6a, b).

### Oleandrin inhibited the protein expression of β-catenin and reduced its nuclear localization

As a key transcriptional factor, the expression and nuclear accumulation of β-catenin directly influenced the activity of the Wnt signaling pathway and regulated the transcription and expression of the target genes. Therefore, we explored the regulating effect of oleandrin on β-catenin by western blot analysis of the total cytoplasmic and nuclear protein extracts. As shown in Fig. 6c, d, oleandrin treatment led to significantly decreased total β-catenin levels over time. According to the nuclear and cytoplasmic protein expression results, we found that β-catenin was located in the cytoplasm as well as in the nucleus of the control. Nevertheless, after treatment with 50 nM of oleandrin, the β-catenin located in nucleus was gradually decreased, and the difference became very evident (*P* < 0.01) after 48 h of treatment. There was a slight decreasing trend in the levels of cytoplamic β-catenin, but no significant difference was observed.

### Effects of oleandrin on the MMP-2 and MMP-9 activities of human OS cells

Gelatin zymography is a simple yet powerful method to detect proteolytic enzymes that are capable of degrading gelatin from various biological sources. It is particularly useful for the assessment of two key members of the matrix metalloproteinase family, MMP-2 and MMP-9 [18, 19]. We used this method to further explore the change of MMP-2 and MMP-9 activities in U2OS cells. With the treatment of 25 nM and 50 nM of oleandrin for 24 h, the ability of MMP-2 and MMP-9 to degrade gelatins was significantly reduced (Fig. 6e).

## Discussion

Osteosarcoma is the most frequent malignant bone tumor and is derived from primitive bone-forming mesenchymal cells with a high propensity for neovascularization and distant metastasis [20], which could seriously threaten the patients’ life.

Recently, oleandrin has been used as a novel drug to treat malignant tumors due to its selectively tumor-killing and radio-chemotherapy sensitization effects. Newman et al. [5] reported that oleandrin had a potent cytotoxicity to human melanoma cells and induced cells apoptosis. Manna et al. [21] reported that oleandrin could suppress the activation of nuclear transcription factor-κB (NF-κB), activator protein-1 (AP-1), and c-Jun NH_2_-terminal kinase. NF-κB and AP-1 are known to play major roles in cell proliferation, tumor promotion, and drug resistance [22]. Boulares et al. [23] also indicated that oleandrin could activate NF-κB in different cell types and induce apoptosis by caspase-dependent PARP cleavage and DNA fragmentation. The study of McConkey et al. [11] demonstrated that oleandrin treatment led to the apoptosis of prostate tumor cells, and this effect is mediated through the inhibition of Na^+^, K^+^-ATPase. Moreover, Frese et al. [7] found that oleandrin could enhance the pro-apoptotic sensibility of non-small cell lung cancer, which is induced by Apo2L/TRAIL through the upregulation of the death receptors 4 and 5. In this study, we explored the effect of oleandrin on OS cells and the related mechanisms.

First, the influence of oleandrin on the viability and proliferation of OS cells were determined by CCK-8 and clone formation assays. Our results showed that oleandrin treatment reduced the viability of U2OS and SaOS-2 cells in a time- and concentration-dependent manner and decreased the cell cloning efficiencies. Under a light microscope, we also observed that following treatment with 25 nM and 50 nM of oleandrin for 24 h, the cell surfaces were irregular and vesicles existed in the cytoplasm, which are typical apoptotic morphological changes [24]. Therefore, we concluded that oleandrin could inhibit the proliferation of OS cells and induce their apoptosis, which was also confirmed by DAPI staining and FCM. DAPI staining showed that oleandrin treatment led to the nuclei of OS cells presenting with pyknotic, karorrhexis and even karyolysis characteristics, while the cell nuclei in the control group were uniformly dispersed. FCM also indicated that the total apoptosis rates of both OS cells were increased significantly with treatment time. All of these findings indicate that oleandrin can dramatically induce OS cell apoptosis, which is consistent with previous studies that reported the apoptosis-induction effect of oleandrin on other tumor cells [25]. Cell migration is a tightly regulated process that occurs in tissue development and underlies pathological conditions, such as cancer invasion, and cell invasiveness, which is a crucial process of cancer metastasis [26, 27]. We also observed the effect of oleandrin on the migration and invasion of U2OS and SaOS-2 cell lines by using a wound healing assay and a transwell invasion assay. The results showed that the migration rates of both cell lines were inhibited and that the number of OS cells that moved from the Matrigel into the substratum of the membrane was significantly decreased by oleandrin application. These results support that oleandrin can not only suppress the migration of OS cells but also inhibit their invasion capacities.

Previous studies had reported that the activation of the Wnt/β-catenin signaling pathway was widely apparent in OS tissue/cells and that its aberrant activation played a significant role in OS tumorigenesis, metastasis and chemotherapeutic responses [28, 29]. In this study, we explored whether oleandrin had an effect on the Wnt/β-catenin signaling pathway, and the dual-luciferase reporter assay with the TOP/FOP flash plasmid system was used to evaluate this mechanism in U2OS cells. The TOP/FOP flash plasmid system is the most common method and has been used by many previous studies to evaluate the transcriptional activity of TCF/LEF in Wnt/β-catenin signaling [30]. In this study, we detected the values of the TOP flash and the FOP flash of OS cells under oleandrin treatment with or without LiCl, an inhibitor of GSK-3β. Additionally, the TOP/FOP flash ratio was presented to reflect the activity of Wnt/β-catenin signaling. The results demonstrate that the TOP/FOP flash ratio dramatically decreased in a time- and concentration-dependent manner, which demonstrates that the transcriptional activity of TCF/LEF was suppressed by oleandrin. Even after the Wnt/β-catenin signaling in OS cells was pre-activated by LiCl, the TOP/FOP flash ratio still declined in a time- and concentration-dependent fashion after exposure to oleandrin. Thus, we speculated that oleandrin may suppress the activity of the Wnt/β-catenin signaling pathway and further influence the downstream genes of this pathway, such as c-myc, cyclin D1, survivin and matrix metalloproteinases (MMPs) [31].

c-Myc is a helix-loop-helix leucine zipper phosphoprotein that regulates gene transcription in cell proliferation, cell differentiation, and programmed cell death [32]. Its overexpression is one of the most common alterations in human cancers. Reports also show that the suppression of c-myc oncogene induces cellular senescence in diverse tumor types, including OS [31]. Survivin is an inhibitor of the apoptosis protein and a key determinant in protecting cells from apoptosis. It is over-expressed in most tumors, such as OS. It displays a significant function on the development of OS and could be taken as a prognostic factor for OS patients [33, 34]. Cyclin D1 is a key regulator of the G1 phase of the cell cycle [35]. It is overexpressed in many cancers, including OS, and regulates cell proliferation through the activation of cyclin-dependent kinases [36]. MMP-2 and MMP-9 are enzymes that are implicated in the malignant progression of many tumor types. They play a vital role in tumor invasion and angiogenesis [37] and are believed to be a critical part of the invasive potential of tumor cells because of their ability to degrade type IV collagen, a major structural component of basement membranes [38]. In OS patients, the overexpression of MMP-2 and MMP-9 is always observed [39].

The mRNA and protein expression of the downstream genes of the Wnt/β-catenin pathway including c-myc, cyclin D1, survivin, MMP-2 and MMP-9 were detected using semi-quantitative RT-PCR and western blot assays. As expected, both assays indicated that oleandrin, at varying treatment lengths, could significantly downregulate the mRNA levels and protein expression of these genes. We already knew that oleandrin inhibited the migration and invasion of OS cells through influencing the expression of MMP-2 and MMP-9. Subsequently, we further explored the change of MMP-2 and MMP-9 activities with gelatin zymography. With the treatment of 25 nM and 50 nM oleandrin for 24 h, the MMP-2 and MMP-9 activities were reduced. These findings demonstrate that oleandrin not only inhibits the expression of MMP-2 and MMP-9 but also suppresses their enzyme activities. Therefore, based on the reports above, we concluded that oleandrin suppressed the Wnt/β-catenin signaling pathway by downregulating the expression of the target genes and also inhibited the enzyme activities of MMP-2 and MMP-9.

β-Catenin is a key molecule in the Wnt/β-catenin signaling pathway, and its shuttling in osteosarcoma cells between the cytoplasm and the nucleus is a common phenomenon in terms of the activation state of the Wnt/β-catenin pathway [40]. In the nucleus, β-catenin interacts with TCF/LEF and triggers the transcription of target genes. Therefore, nuclear β-catenin accumulation is a key event in the activation of Wnt/β-catenin signaling. The deregulation of the Wnt/β-catenin pathway would result in the aberrant accumulation of β-catenin in the nucleus, which occurs in parallel to the development of cancer [41]. In this study, we found that the levels of total and nuclear β-catenin were significantly decreased in response to varying treatment lengths. Although there was a slight decreasing trend in the levels cytoplasmic β-catenin, no significant difference was observed. These results indicate that oleandrin treatment could effectively reduce the nuclear location of β-catenin. To a certain degree, our findings are consistent with those of previous studies, which have reported that the suppression of the Wnt/β-catenin signaling pathway would lead to the reduction of nuclear β-catenin [42]. Our results further demonstrated that oleandrin could suppress the activity of the Wnt/β-catenin signaling pathway.

Of course, there were limitations to this study. We only explored the antitumor effect and underlying mechanism of oleandrin on two human OS cell lines in vitro. Hence, in our further studies, we will investigate the antitumor effect of oleandrin in animal models and we will study the detailed mechanisms of the invasion inhibition and apoptosis-inducing effects of oleandrin in vivo.

## Conclusions

In conclusion, we found that oleandrin, in vitro, could inhibit proliferation, induce apoptosis and reduce the invasiveness of U2OS and SaOS-2 cells. The antitumor activities of oleandrin on OS cells were probably achieved by suppressing the Wnt/β-catenin signaling pathway, which resulted in the down-regulation of target genes and decreased the total and nuclear β-catenin. In addition, oleandrin not only reduced MMP-2 and MMP-9 expression but also suppressed their activities.

## Abbreviations

OS: Osteosarcoma
TCF/LEF: T-cell factor/lymphocyte enhancer factor
MMPs: Matrix metalloproteinase
DAPI: Dye 4’-6-diamidino-2-phenylindole
NF-κB: Nuclear transcription factor-κB
AP-1: Activator protein-1
GSK 3β: Glycogen synthase kinase 3β
APC: Adenomatous polyposis coli
Fz: Frizzled
LRP5/6: Lipoprotein receptors 5/6
Dsh: Disheveled
FCM: Flow cytometry
PI: Propidium iodide
SD: Standard deviation
ANOVA: Analysis of variance
